# Which injured patients with moderate fibrinogen deficit need fibrinogen supplementation?

**DOI:** 10.1186/s13049-021-00988-x

**Published:** 2021-12-24

**Authors:** Jean-Stephane David, Aline Lambert, Xavier-Jean Taverna, Pascal Incagnoli, Marie-Odile Geay-Baillat, Olivia Vassal, Arnaud Friggeri, Kenji Inaba

**Affiliations:** 1 Service d’anesthésie-réanimation, Centre Hospitalier Lyon Sud, Hospices Civils de Lyon, 69495 Pierre Benite, France; 2 Faculté de Médecine Lyon Est, Université Claude Bernard Lyon 1, Lyon, France; 3 Research on Healthcare Performance (RESHAPE), INSERM U1290, Université Claude Bernard Lyon 1, Lyon, France; 4 Service d’anesthésie-réanimation, Hôpital Edouard Herriot, Hospices Civils de Lyon, 69008 Lyon, France; 5 Service des Urgences – SAMU, Hôpital Universitaire de Dijon, Dijon, France; 6 Laboratoire d’Hémostase, Hôpital Lyon Sud, Hospices Civils de Lyon, 69495 Pierre Benite, France; 7 Division of Trauma and Critical Care, Department of Surgery, LAC + USC Medical Center, University of Southern California, Los Angeles, CA USA; 8 Service d’Anesthésie Réanimation, Centre Hospitalier Lyon Sud, 69495 Pierre Bénite cedex, France

**Keywords:** ROTEM, Fibrinogen, Trauma, Coagulopathy, Shock

## Abstract

**Background:**

In severely injured patients, fibrinogen supplementation is recommended when fibrinogenemia is < 1.5 g L^−1^, but some teams have suggested to use higher thresholds (fibrinogenemia < 2.0 g L^−1^ or FIBTEM clot amplitude at 5 min (A5) values < 11 mm). The goal of this study was to specify in patients with a moderate fibrinogen deficit (MFD) whether some admission characteristics would be associated with fibrinogen administration at 24 h.

**Methods:**

Prospective analysis of retrospectively collected data from a trauma registry (01/2011–12/2019). MFD-C was defined by a fibrinogenemia 1.51–1.99 g L^−1^ or the corresponding FIBTEM-A5 values (MFD-A5) that were determined from linear regression and ROC curve analysis. Administration of fibrinogen were described according to the following admission parameters: shock index (SI) > 1, hemoglobin level < 110 g L^−1^ (HemoCue®), and base deficit > 5 mEq L^−1^. Data are expressed as count (%), median [IQR].

**Results:**

1076 patients were included in the study and 266 (27%) had MFD-C, among them, 122/266 (46%) received fibrinogen. Patients with MFD-C who received fibrinogen were more severely injured (ISS: 27 [19–36] vs. 24 [17–29]) and had more impaired vital signs (base deficit: 5.4 [3.6–7.8] vs. 3.8 [2.0–6.0]). Linear regression analysis found a positive correlation between fibrinogen level and FIBTEM-A5 (r: 0.805). For a fibrinogen level < 1.5 g L^−1^ and < 2.0 g L^−1^, FIBTEM-A5 thresholds were 6 mm (sensitivity 85%, specificity 83%, AUC: 0.934) and 9 mm (sensitivity 84%, specificity 69%, AUC: 0.874), respectively. MFD-A5 values (185 (27%) patients) were defined as a FIBTEM-A5 between 7 and 9 mm. More than 50% of MFD-C patients presenting a SI > 1, a hemoglobin level < 110 g L^−1^, or a base deficit > 5.0 mEq L^−1^ received fibrinogen. The relative risk [95% CI] for fibrinogen administration (SI > 1) were 1.39 [1.06–1.82] for MFD-C, and 2.17 [1.48–3.19] for MFD-A5. Results were not modified after adjustment on the ISS.

**Conclusions:**

We have shown in this study an association between shock parameters and fibrinogen administration. Further studies are needed to determine how these parameters may be used to guide fibrinogen administration in trauma patients with MFD.

## Introduction

Coagulopathy is frequently observed among critically-injured patients and is estimated to occur in 20–30% of patients at admission [1–3]. The early identification and treatment of trauma-induced coagulopathy (TIC) is critical for optimizing outcomes [4]. For over a decade now, it has been suggested that viscoelastic techniques (VET) be used to provide a rapid assessment of hemostatic disorders, of which fibrinogen deficit is the most frequently observed [3, 5]. All of these abnormalities are further exacerbated by shock and hypoperfusion [6].

European Guidelines suggest fibrinogen deficit to be treated when the fibrinogen level is less than 1.5 g L^−1^ in injured patients with ongoing bleeding. However, the potential benefit of earlier fibrinogen supplementation with fibrinogen concentrates or cryoprecipitate remains debated. For example, it has been suggested by some teams that fibrinogen should be administered when the level is less than 2.0 g L^−1^ or when the FIBTEM clot amplitude at 5 min is less than 11 mm [7, 8]. While the use of higher fibrinogen thresholds may help to decrease the bleeding by increasing the clot firmness, it may also lead to unnecessary administration of fibrinogen, increased costs or, more importantly, to adverse pro-coagulant events. It is therefore important during the resuscitation of injured patients to define precisely which patients, with a moderate fibrinogen deficit (MFD), will benefit from fibrinogen supplementation.

The objective of this study was to describe the characteristics of patients with moderate fibrinogen deficiency at admission, determined using the Clauss technique (MFD-C) or by thromboelastometry (MFD-A5), and to specify in this group of patients which criteria were associated with the administration of fibrinogen.

## Methods

### Design and setting

This was a retrospective analysis of data of severely injured patients > 15 years old who were admitted to the trauma resuscitation unit of a university hospital from January 1, 2011, to December 31, 2019. This study was reported according to the STROBE Statement for observational studies. The data used in the study were retrieved from a prospectively built registry. The rate of missing data was low and consisted primarily of prehospital vital signs. The most frequently missing data during the prehospital phase of care was for heart rate (117 patients, 11%) and fluid volume (108 patients, 10%). No missing data was observed for the vital signs at admission, ISS, outcomes or blood products.

The regional emergency network RESUVAL supervised the registry and obtained official approval from the *Commission Nationale Informatique et Liberté* (DE 2012-059), the CCTIRS (*Comité consultatif sur le traitement de l'information en matière de recherche*), and the Institutional review board (02/2020). Written informed consent was not required and all patients or their next of kin were provided with information about the registry.

All patients, as is usual practice in France, were cared for and triaged during the prehospital phase by a physician who can be an anesthesiologist or an emergency medicine physician (‘SAMU system’) [9].

### Population selection

Patients were included in the study protocol if they met at least one of the following criteria: (1) received during the first 24 h after admission at least one unit of blood product or coagulation factor concentrate, (2) had a ROTEM analysis, (3) were admitted to a critical care unit. Patients were excluded if they (1) were treated with anticoagulants, (2) received plasma or platelet concentrates during the prehospital phase, (3) were transferred from another hospital or did not have hemostasis analysis at admission (ROTEM or standard hemostasis testing). For each patient, we recorded the demographic and injury characteristics including the Injury Severity Score (ISS), as well as intensive care unit length of stay and survival at 24 h and at hospital discharge.

### Laboratory analyses

Blood samples were collected at admission by venipuncture into Vacutainer tubes (Becton Dickinson, Plymouth, UK) containing citrate (0.129 M trisodium citrate) for standard tests (Star Evolution; Diagnostica Stago, Asnieres, France): prothrombin time (PT) (STA-Neoplastin CI plus), activated partial thromboplastin time (STA-PTT automat), fibrinogen (Clauss technique, STA-Fibrinogen), and/or thromboelastometry (Werfen, Le Pré-Saint-Gervais, France). The choice of performing standard laboratory testing or ROTEM® analysis at admission or during follow-up was made at the discretion of the attending physician.

The ROTEM® coagulation analyser (Delta, Werfen, Munich, Germany) has been described previously in detail [10, 11]. In the ROTEM® analyser, coagulation is partially activated with recombinant human tissue factor (EXTEM test). In addition to the EXTEM screening tests, cytochalasin D (FIBTEM) was used to study the EXTEM with the inhibition of platelets to assess fibrin polymerization. The ROTEM® analysis was performed at 37 °C. Among the ROTEM® parameters, the FIBTEM clot amplitude at 5 min (A5) was analysed for this study. ROTEM® analyses were performed following standard and recommended procedures throughout the study in the local hemostasis laboratory where the ROTEM® device was located. The results were immediately transferred and available on a computer located in the trauma resuscitation unit.

Hemoglobin measurement using the HemoCue® device was performed at hospital admission on capillary blood in hemodynamically stable patients, whereas it was performed for shocked patients on venous or arterial blood, as suggested by the unit protocol.

### Blood products administration

During the first 24 h after admission, all the blood products administered to the patients were registered. Treatment options for patients with coagulopathy were fibrinogen concentrates (Clottafact, LFB laboratory, Les Ullis, France) for a fibrinogen deficit, fresh frozen plasma and/or prothrombin complex concentrate (PCC, Kanokad, LFB laboratory, Les Ullis, France) for a coagulation factor deficit, and platelet concentrate for thrombocytopenia. While the treatment decision was ultimately made at the discretion of the attending physician, both French and European guidelines were followed and fibrinogen concentrates were administered if the fibrinogen was < 1.5 g L^−1^, or if there was evidence of a fibrinogen deficit on the ROTEM analysis (FIBTEM-A5 < 7 mm) [4, 12]. FFP and/or PCC were administered if the PT was less than 40% (i.e. PT_ratio_ > 1.8) [13], or if there was evidence of a coagulation factor deficit (EXTEM CT > 90 s and FIBTEM-A5 > 6 mm or EXTEM CT > 106 s) [12]. Platelet concentrates were administered if the platelet count was less than 50 × 10^9^ L^−1^ or 100 × 10^9^ L^−1^ in case of hemorrhagic shock or severe brain injury [4]. Patients were administered tranexamic acid either during the prehospital phase of care or at admission, according to French and European guidelines [4, 14].

### Statistical analysis

Baseline characteristics were expressed as count (percentages) for categorical variables and for continuous variables, with median [interquartile range, IQR] or mean ± standard deviation according to the normality of the distribution that was tested with a Kolmogorov–Smirnov test. The differences between study groups were analyzed using the Kruskal–Wallis one-way analysis of variance rank test with a Bonferroni post hoc test or for qualitative variables, with the Pearson’s x^2^ test. A *p* value < 0.05 was considered as statistically significant.

Linear regression analysis was performed to describe the relationship between FIBTEM-A5 and Clauss fibrinogen, and the Spearman coefficient was determined.

For the study, we defined 3 groups of patients according to the Clauss fibrinogen measurements: severe fibrinogen deficit (fibrinogen < 1.50 g L^−1^), moderate fibrinogen deficit (MFD-C: fibrinogen ≥ 1.50 and ≤ 1.99 g L^−1^) and no fibrinogen deficit (fibrinogen ≥ 2.00 g L^−1^). The FIBTEM-A5 (MFD-A5) values corresponding to 1.50 and 1.99 g L^−1^ with a sensitivity> 80% were determined from the Receiver Operating Characteristic (ROC) curves and the area under the ROC curve (AUC) was calculated.

We studied the relationship of several parameters immediately available or measured at admission (systolic blood pressure, shock index, base deficit, lactate level, and point-of-care hemoglobin (Hemocue)), with the need for fibrinogen replacement during the first 24 h. ROC curves were drawn for each parameter, the AUCs were calculated and compared using the De Long test (systolic blood pressure vs. shock index; lactate vs. base deficit). The parameter with the best AUC was then used for analysis with the thresholds previously described in the literature (for example, shock index > 1, base deficit > 5, and hemoglobin < 110 g.dL^−1^) [15–17]. For each of the previous parameters, the relative risk was calculated and reported with its 95% confidence interval (95% CI).

All statistical tests were performed using commercially available statistical software (NCSS 9.0.22, Kaysville, Utah; Medcalc 9.3.6.0, Mariakerke, Belgium).

## Results

**Fig. 1:**
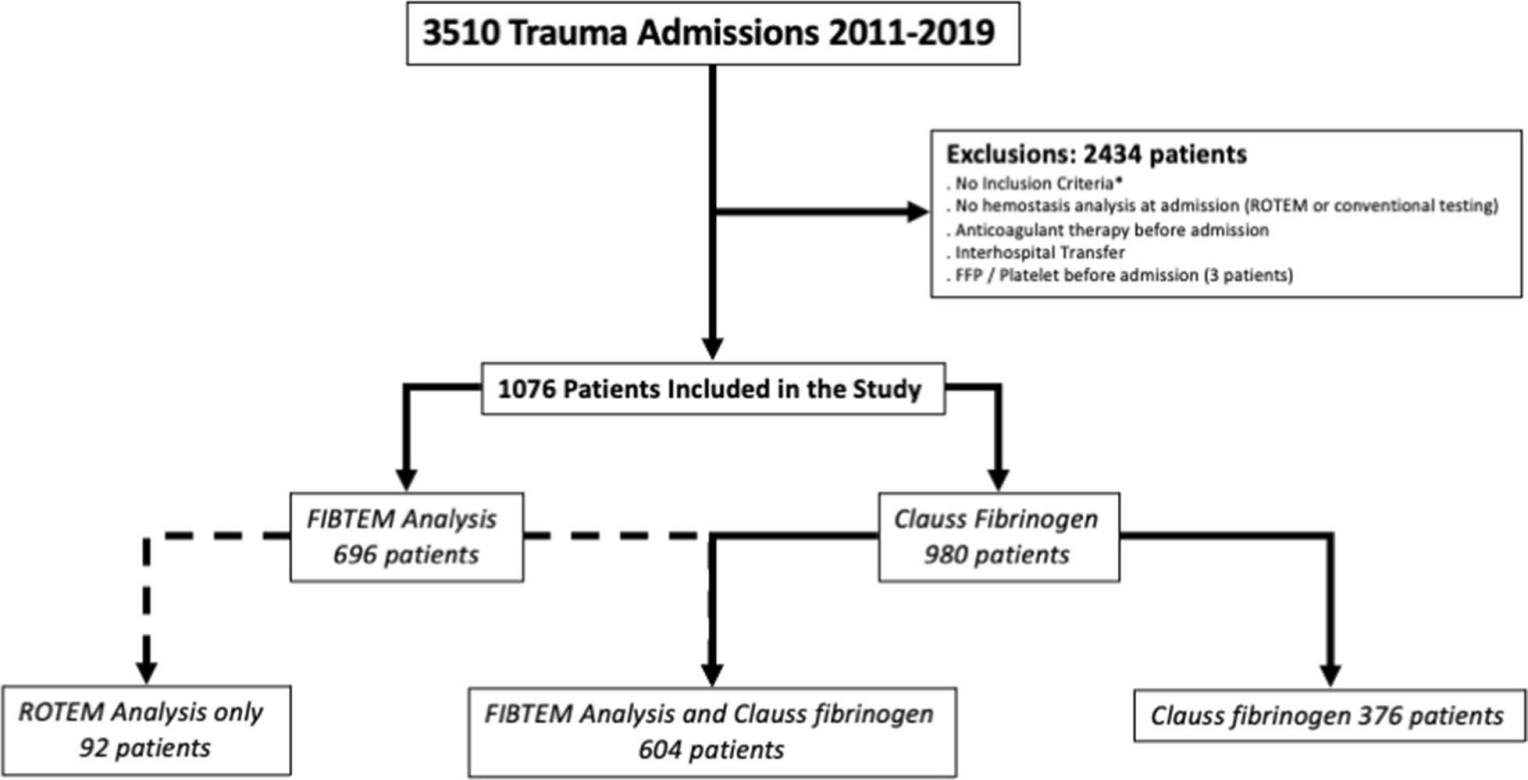
Flowchart of the study. *Inclusion criteria: (1) having a ROTEM analysis performed at admission, and/or (2) receiving blood products/coagulation factor concentrates during the first 24 h after admission, and/or (3) being admitted in an intensive care unit

From January 1, 2011, to December 31, 2019, a total of 3510 patients were admitted in the study center and 1076 (31%) of them met the inclusion criteria and were therefore included in the study (Fig. 1).

Baseline and demographic characteristics, blood product administration and laboratory analysis of patients for whom Clauss fibrinogen level was determined at admission are detailed in Table 1.

**Table 1:** Demographic and injury characteristics of patients for whom Clauss fibrinogen level was determined at admission

Study group	All patients	Severe deficit	Moderate deficit	No deficit	*p*
Fibrinogenemia (g L^−1^)		< 1.50	1.51–1.99	> 1.99	
N	980	158 (16)	266 (27)	556 (57)	
Demographic data					
Age (years)	41 [25–57]	38 [24–53]	31 [22–50]	46 [28–60]	< 0.001
Sex male	745 (76)	118 (75)	207 (78)	420 (76)	0.705
Blunt Trauma	918 (94)	150 (95)	248 (93)	520 (94)	0.766
ISS	25 [17–33]	38 [28–50]	25 [18–33]	22 [16–29]	< 0.001
Mortality at discharge	188 (19)	81 (51)	43 (16)	64 (12)	< 0.001
Prehospital parameters					
GCS	14 [6–15]	5 [3–14]	14 [8–15]	14 [9–15]	< 0.001
SBP (mmHg)	120 [100–140]	95 [60–123]	120 [100–136]	127 [110–144]	< 0.001
Shock Index	0.7 [0.6–1.0]	1.1 [0.7–1.6]	0.8 [0.6–1.0]	0.7 [0.6–0.8]	< 0.001
Fluids (mL)	850 [500–1250]	1500 [1000–2213]	1000 [500–1500]	750 [350–1000]	< 0.001
Vasopressor	200 (20)	89 (56)	41 (16)	70 (13)	< 0.001
Admission parameters					
SBP (mmHg)	118 [100–136]	101 [73–124]	115 [99–133]	122 [108–140]	
Shock Index	0.7 [0.6–0.9]	1.0 [0.7–1.3]	0.8 [0.6–1.0]	0.7 [0.6–0.8]	< 0.001
HemoCue < 110 g L^−1^	248 (26)	102 (66)	67 (26)	79 (15)	< 0.001
Lactate (mmol L^−1^)	2.3 [1.5–3.7]	4.4 [2.6–7.8]	2.4 [1.7–3.5]	2.0 [1.3–2.9]	< 0.001
Base deficit (mEq L^−1^)	4.6 [2.2–7.5]	10.7 [6.3–16.2]	4.7 [2.6–7.0]	3.5 [1.4–5.7]	< 0.001
Hemoglobin (g L^−1^)	126 [109–139]	94 [78–116]	126 [110–137]	132 [119–143]	< 0.001
PT_ratio_	1.1 [1.1–1.3]	1.6 [1.3–2.2]	1.2 [1.1–1.3]	1.1 [1.0–1.1]	< 0.001
Fibrinogen (g L^−1^)	2.1 [1.7–2.6]	1.0 [0.6–1.3]	1.8 [1.6–1.9]	2.5 [2.2–3.0]	< 0.001
Platelet (10^9^ L^−1^)	214 [179–259]	173 [121–204]	220 [183–266]	225 [190–268]	< 0.001
Blood products [24 h]					
Fibrinogen concentrate (g)	0.0 [0.0–1.5]	4.5 [3.0–6.0]	0.0 [0.0–3.0]	0.0 [0.0–0.0]	< 0.001
Red blood cell (unit)	0 [0–2]	3 [1–6]	0 [0–2]	0 [0–0]	< 0.001
Fresh frozen plasma (unit)	0 [0–0]	1 [0–4]	0 [0–0]	0 [0–0]	< 0.001
Platelet (unit)	0 [0–0]	0 [0–3]	0 [0–0]	0 [0–0]	< 0.001
TXA first 3 h*	679 (70)	137 (87)	225 (85)	317 (57)	< 0.001

Data are expressed as count (percentage) or median [interquartile range]. Mortality is at hospital discharge

*SBP* systolic blood pressure, *ISS* injury severity score, *TXA* tranexamic acid

*TXA administered during the first 3 h following the injury

During the prehospital phase of care, 232 (22%) patients received vasopressor and 49 (5%) patients received a mean of 1.8 units of red blood cells. At admission, 328 (33%) patients had a PT_ratio_ > 1.20. A ROTEM® analysis with a FIBTEM measure was performed for 696 (65%) patients. The median [IQR] length of stay in the intensive care unit was 3 [0–10] days and the mortality rate at hospital discharge was 20% (212/1076 patients).

### Determination of threshold FIBTEM-A5 values corresponding to Clauss fibrinogen levels 1.50 and 2.00 g L^−1^

At admission, 158 (16%) patients had a fibrinogen level < 1.50 g L^−1^ and 424 (43%) a fibrinogen level < 2.00 g L^−1^ (Table 1).

**Fig. 2:**
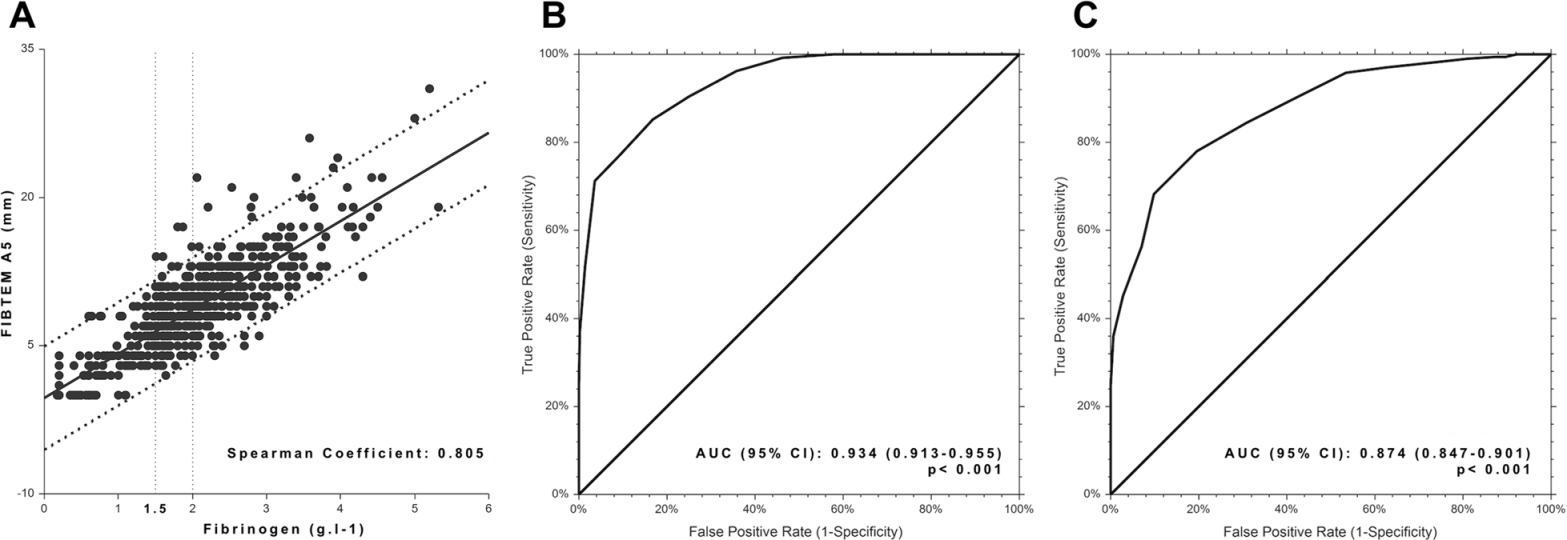
Linear regression between the Clauss fibrinogen levels and FIBTEM-A5 values (Panel **A**), and ROC curve analysis for the prediction of a fibrinogenemia < 1.50 g L^−1^ (Panel **B**) and < 2.00 g L^−1^ (Panel **C**) in 604 patients

The linear regression analysis found a very good correlation between Clauss fibrinogen level and FIBTEM-A5 values. The FIBTEM-A5 values [95% CI] corresponding to 1.5 and 2.0 g L^−1^ were 6.4 [6.2–6.7] mm and 8.7 [8.5–8.9] mm, respectively. For fibrinogen levels < 1.50 g L^−1^, the ROC curve analysis demonstrated that a FIBTEM-A5 threshold value of 6 mm was associated with a sensitivity [95% CI] of 85% [78–91] and specificity [95% CI] of 83% [79–86]. For fibrinogen levels < 2.00 g L^−1^, the ROC curve analysis demonstrated that a FIBTEM-A5 threshold value of 9 mm was associated with a sensitivity [95% CI] of 84% [80–88] and specificity [95% CI] of 69% [64–75] (Fig. 2). With these 2 threshold values, MFD-A5 was defined as a FIBTEM clot amplitude between 7 and 9 mm.

### Fibrinogen administration according to fibrinogen or FIBTEM levels

Fibrinogen concentrates were administered to 122/266 (46%) patients with MFD-C, 142/158 (90%) patients with severe fibrinogen deficit, and 37/556 (7%) patients without fibrinogen deficit (*p* < 0.001). In the group of patients with MFD-A5, 61/185 (33%) patients received fibrinogen supplementation during the first 24 h after admission, whereas 188/224 (84%) patients with a severe fibrinogen deficit (FIBTEM-A5 < 7 mm) and 28/287 (9.8%) patients with no fibrinogen deficit (FIBTEM-A5 > 9 mm) did (*p* < 0.001).

On 266 patients with MFD-C, 65 patients (24%) received fibrinogen and RBC, 57 patients (21%) received fibrinogen, 22 patients received RBC (8%) and 122 patients (46%) received nothing. Patients with MFD-C who received fibrinogen, as compared to those that did not receive fibrinogen, were more severely injured, had more impaired vital signs at admission (Table 2) and had higher mortality at hospital discharge (26 patients (21%) vs. 17 patients (12%), *p*: 0.036). Similar differences were observed in the group of patients with MFD-A5 (data not shown).

**Table 2:** Characteristics of MFD-C patients according to critical parameters at admission and fibrinogen administration at 24-h

Study group	Shock index > 1.0		Hemoglobin < 110 g L^−1^		Base deficit > 5.0 mEq L^−1^		Fibrinogen administration	
	No	Yes	No	Yes	No	Yes	No	Yes
N	211 (80)	54 (20)	195 (74)	67 (26)	133 (56)	106 [44]	144 (54)	122 (46)
Demographic data								
Age (years)	31 [22–50]	28 [22–46]	28 [21–45]	42* [25–65]	29 [21–49]	35 [23–50]	33 [22–50]	28 [22–46]
ISS	25 [18–33]	29 [17–38]	24 [17–30]	27* [18–41]	24 [17–29]	29* [22–38]	24* [17–29]	27 [19–36]
Prehospital parameters								
GCS	14 [8–15]	14 [7–15]	15 [9–15]	14* [7–15]	14 [9–15]	13* [6–15]	14 [10–15]	14 [6–15]
SBP (mmHg)	122 [104–137]	115 [96–130]	123 [108–138]	110* [80–132]	125 [108–140]	114* [94–131]	123 [105–138]	118 [100–134]
Fluids (mL)	1000 [500–150]	1375* [1000–1563]	1000 [500–1500]	1250* [1000–1500]	750 [500–1250]	1250* [750–1500]	1000 [500–1500]	1000 [600–1500]
Admission parameters								
SBP (mmHg)	121 [108–135]	90* [77–102]	120 [103–134]	101* [87–123]	120 [107–135]	111* [90–132]	116 [101–133]	116 [95–134]
Shock Index	0.7 [0.6–0.9]	1.3* [1.1–1.5]	0.8 [0.6–1.0]	0.9* [0.7–1.1]	0.7 [0.6–0.9]	0.9* [0.7–1.1]	0.8* [0.6–1.0]	0.8 [0.7–1.1]
Base deficit (mEq L^−1^]	4.3 [2.3–6.1]	6.9* [4.6–10.8]	3.8 [2.3–5.8]	6.9* [4.7–8.9]	2.8 [1.9–3.9]	7.3* [5.9–9.2]	3.8* [2.0–6.1]	5.4 [3.6–7.8]
Hemoglobin (g L^−1^)	127 [113–140]	115* [93–126]	129 [121–141]	94* [86–106]	127 [116–141]	119* [101–131]	127* [115–140]	120 [101–133]
PT_ratio_	1.2 [1.1–1.3]	1.2* [1.1–1.4]	1.1 [1.1–1.2]	1.3* [1.2–1.4]	1.1 [1.1–1.2]	1.2* [1.1–1.3]	1.1* [1.1–1.2]	1.2 [1.1–1.3]
Fibrinogen (g L^−1^)	1.8 [1.6–1.9]	1.7* [1.6–1.8]	1.8 [1.6–1.9]	1.8 [1.6–1.9]	1.8 [1.6–1.9]	1.7* [1.6–1.8]	1.8* [1.7–1.9]	1.7 [1.6–1.8]
Platelet (10^9^ L^−1^)	219 [184–258]	226 [183–290]	225 [188–271]	208* [154–246]	221 [188–258]	213 [175–277]	225 [189–271]	210 [179–255]
Red blood cell (unit)	0.0 [0.0–0.0]	1.5* [0.0–3.0]	0.0 [0.0–0.0]	2.0* [0.0–4.0]	0.0 [0.0–0.0]	0.0* [0.0–2.0]	0.0* [0.0–0.0]	1.0 [0.0–3.0]
Fibrinogen concentrate								
(g)	0.0 [0.0–0.0]	1.5* [0.0–3.0]	0.0 [0.0–0.0]	2.0* [0.0–3.0]	0.0 [0.0–2.0]	1.5* [0.0–3.0]	–	3.0 [1.5–3.0]
N (%)	90 (43)	32* (59)	78 (40)	42* (63)	47 (35)	63* (59)	–	–

Data are expressed as count (percentage) or median [interquartile range]

*SBP* systolic blood pressure, *ISS* injury severity score, *PT*_*ratio*_ prothrombin time ratio

*p < 0.05 for the difference between yes versus no.

We observed that 38 patients (14%) with MFD-C and without sign of shock at admission received nevertheless fibrinogen (median [IQR]: 3.0 g [1.5–3.0]) among those, 15 patients (39%) also received RBC (median [IQR]: 0 unit [0–2]). Among these, 31 patients underwent urgent surgery within hours after admission, 2 patients with active bleeding visualized on whole body-CT underwent angioembolization, and for 5 patients, the indication for fibrinogen administration was the presence of severe injury with a significant bleeding risk.

### Relationship between critical parameters measured at admission and fibrinogen administration

In the whole study cohort, for the prediction of fibrinogen administration, the shock index AUC was significantly higher compared to the systolic blood pressure AUC (0.698, 95% CI [0.662–734] vs. 0.668, 95% CI [0.631–705], *p*: 0.049); similarly the base deficit AUC was significantly higher compared to the lactate AUC (0.767, 95% CI [0.735–0.799] vs. 0.718, 95% CI [0.682–0.753], *p*: 0.0007). Both the shock index and base deficit were then used for final analysis. The hemoglobin (HemoCue®) value AUC to predict fibrinogen administration was 0.742, 95% CI [0.709–775].

MFD-C patients presenting a shock index > 1, a hemoglobin level < 110 g L^−1^, or a base deficit > 5 mEq L^−1^ at admission were more severely injured (ISS) and were more in proportion to require fibrinogen concentrate and red blood cell transfusion (Table 2). In univariate analysis, the studied parameters (shock index > 1, hemoglobin level < 110 g L^−1^, and base deficit > 5 mEq L^−1^) were associated with fibrinogen supplementation at 24 h in MDF-C patients (Table 3). These results persisted after adjustment on the ISS (data not shown). When one of these parameters was present at admission, more than 50% of patients received fibrinogen supplementation during the first 24 h.

**Table 3:** Univariate analysis describing the association of admission parameters and fibrinogen replacement in the group of patients with moderate fibrinogen deficit

	Relative risk	[95% CI]	*p*
Fibrinogen 1.50–1.99 g L^−1^ (N = 266)			
Shock index > 1.0	1.39	[1.06–1.82]	0.017
Hemoglobin level < 110 g L^−1^	1.57	[1.22–2.02]	< 0.001
Base deficit > 5.0 mEq L^−1^	1.68	[1.27–2.22]	0.003
FIBTEM-A5 7–9 mm (N = 185)			
Shock Index > 1.0	2.17	[1.48–3.19]	< 0.001
Hemoglobin < 110 g L^−1^	2.43	[1.64–3.60]	< 0.001
Base deficit > 5.0 mEq L^−1^	2.56	[1.59–4.11]	< 0.001

*95% CI* 95% confidence interval. Hemoglobin level was determined at admission with a point-of-care device (HemoCue®)

The same results were observed for patients with MFD-A5 (Table 3). When one of these critical parameters was present at admission, more than 50% of patients with MFD-A5 received fibrinogen supplementation during the first 24 h.

## Discussion

In this study, we demonstrated a very good correlation between Clauss fibrinogen levels and FIBTEM-A5 values, establishing FIBTEM-A5 cut-off values that define a moderate fibrinogen deficit with ROTEM. We observed that patients with moderate fibrinogen deficit who received fibrinogen were more severely injured, displayed more impaired vital signs and laboratory abnormalities. At admission, shock index > 1, hemoglobin level < 110 g L^−1^, and base deficit > 5.0 mEq L^−1^ were associated with fibrinogen administration in patients with a moderate fibrinogen deficit.

In the first part of the study, we observed that Clauss fibrinogen levels correlated well with FIBTEM-A5 values, as previously described [18–21]. We suggested corresponding threshold values for Clauss fibrinogen level of 1.50 and 2.00 g L^−1^, whereas previous reports have focused on only one value or the other [7, 8, 20, 22]. Rather than defining one threshold value to guide the administration of fibrinogen in patients with moderate fibrinogen deficit, we suggest the incorporation of the clinical evaluation to the Clauss or FIBTEM assay. Rapidly correcting TIC is an important objective during the initial care of severely injured patients, and it has been shown that early correction was associated with improved outcomes [4]. From recent European guidelines, in case of ongoing bleeding, it has been recommended to maintain fibrinogen levels above 1.5 g L^−1^, but some teams have recommended a higher trigger of 2.0 g L^−1^ [7, 8]. If fibrinogen supplementation is recommended for values below 1.5 g L^−1^, but not provided for values above 2.0 g L^−1^, there remains a grey zone between 1.5 and 2.0 g L^−1^, for which some patients may benefit from fibrinogen administration. Using a cut-off value of 2.0 g L^−1^ has been suggested even though it may lead to an increase in the rate of fibrinogen administration. We observed in this study that less than 50% of the patients with a moderate deficit finally had fibrinogen supplementation administered at 24 h. Hence, administering fibrinogen to injured patients based only on standard laboratory or viscoelastic values can lead to unnecessary use, which is related to potential adverse events and increased cost. We observed that patients who had received fibrinogen were more severely injured and displayed a higher mortality rate. This relationship between shock, tissue hypoperfusion, and increased blood requirement has been previously reported [4, 6]. For example, Mutschler et al. [23] have reported a close relationship between worsening shock index and blood requirements, and Hsu et al. [24] have shown a relationship between base deficit and massive transfusion. To ensure a more rational approach to fibrinogen administration, we recommend using parameters that are rapidly available at the time of patient admission and that are widely used across trauma centers. Using these parameters may help clinicians to decide on the administration of fibrinogen while mitigating waste. When at least one of these parameters was present in this study, we observed that fibrinogen administration was required for more than 50% of the patients with a moderate fibrinogen deficit. If the patient with a moderate deficit does not present any of the parameters previously described, the Clauss or FIBTEM test can be repeated, depending on the patient’s clinical condition. If the bleeding has stopped, and the fibrinogen has corrected itself at 24 h, in studies comparing the administration of fibrinogen with placebo, no difference was observed in terms of outcomes [25, 26].

### Study limitations

The limitations of this study include the potential selection bias inherent to all retrospective studies. The patient population analyzed did not include consecutive trauma patients. However, all the most severely injured patients and all of those who received fibrinogen were included in the study. Secondly, we included patients who had physician-based prehospital care and as such, our results may not directly apply in a system in which care is ensured by non-physician providers, or when the transportation time is very short. Prehospital care included fluid infusion that have the potential to induce dilution with a concomitant fibrinogen decrease or clot impairment [27]. The cohort of patients with MFD-C received a mean of 1069 mL of fluids, which is likely insufficient to induce significant hemodilution but should be noted as a potential limitation [28]. Thirdly, these results may not apply to patients with penetrating trauma since this mechanism is infrequent in the studied trauma population (6%) [29]. For example, only 18 patients with a penetrating trauma had a moderate Clauss fibrinogen deficit. However, penetrating mechanism was not associated with fibrinogen administration in this subgroup of patients or even in the whole cohort. A final limitation point concerns the administration of fibrinogen in 38 patients with MFD-C without obvious ongoing bleeding or sign of shock at admission. To date, there are only little evidence suggesting that increasing clot firmness may help to decrease bleeding and hence, the use of RBC in non-bleeding patients. Past European guidelines suggested to give fibrinogen if fibrinogen level was < 1.5 g L^−1^ and if it was associated with a significant bleeding [30]. However, bleeding and coagulopathy are dynamic phenomenon that may be exacerbated by ongoing resuscitation and surgical procedures. Hence, most patients in this subgroup had a surgical procedure and when we looked at the lowest hemoglobin and fibrinogen values during the first 24-h following admission in this subgroup of patients, we observed that they were significantly lower as compared to the admission value ((mean ± SD), hemoglobin: 98 ± 14 vs. 130 ± 14 g L^−1^, *p* < 0.001; fibrinogen: 1.5 ± 0.2 vs. 1.7 ± 0.1, *p* < 0.001), thus attesting to significant bleeding.

## Conclusion

In this study, we showed an association between shock parameters and fibrinogen administration. Further studies are needed to determine how these parameters may be used to guide fibrinogen administration in trauma patients with moderate hypofibrinogenemia.

## Data Availability

Data are available upon request due to privacy or other legal restrictions. Please contact authors for data requests.
